# An Undescribed Monteggia Type 3 Equivalent Lesion: Lateral Dislocation of Radial Head with Both-Bone Forearm Fracture

**DOI:** 10.1155/2016/8598139

**Published:** 2016-04-03

**Authors:** Adnan Kara, Mahmut Enes Kayaalp, Mehmet İşyar, Cem Sever, Melih Malkoç, Mahir Mahiroğulları

**Affiliations:** ^1^Department of Orthopaedics and Traumatology, Istanbul Medipol University School of Medicine, Bagcilar, 34214 Istanbul, Turkey; ^2^Department of Orthopaedics and Traumatology, Istanbul University Cerrahpasa Faculty of Medicine, Fatih, 34098 Istanbul, Turkey

## Abstract

Monteggia fractures are accepted as hard-to-recognize and easy-to-handle fractures. Adequate radiographic investigations and clinical examinations are necessities. This case holds unique features involving diagnosis and treatment. In this case, the radial head was dislocated laterally while both bones were fractured in the proximal diaphysis, being the first to be mentioned in the literature. Closed reduction of the ulna is the preferred method of handling and almost always results in reduction of the radial head. Literature obligates ulnar reduction as a preliminary to reduce and stabilize the radial head. Closed reduction reduced the ulna but the radial head was not reduced. Hence an intramedullary K-wire was used to reduce the radial head and a long arm cast was used to stabilize the reduction. The operation was successful and follow-up showed no complications.

## 1. Introduction 

A Monteggia fracture is by definition a radial head dislocation with a concomitant ulnar fracture.

Obtaining as well as interpreting adequate and good-quality radiographs properly is the most important step in successful recognition and treatment of Monteggia fractures. Preventing delayed diagnosis is a major concern.

Bado classified four subtypes while adding another subtype group as Monteggia equivalent injuries. There are many types mentioned in the literature. However, there was no case reporting any lateral dislocation of the radial head with concomitant fracture of both bones.

Treatment with a radial implantation only, although literature suggests preliminary ulnar fixation, was also successful in our case without any complication.

## 2. Case Presentation 

**Figure 1:**
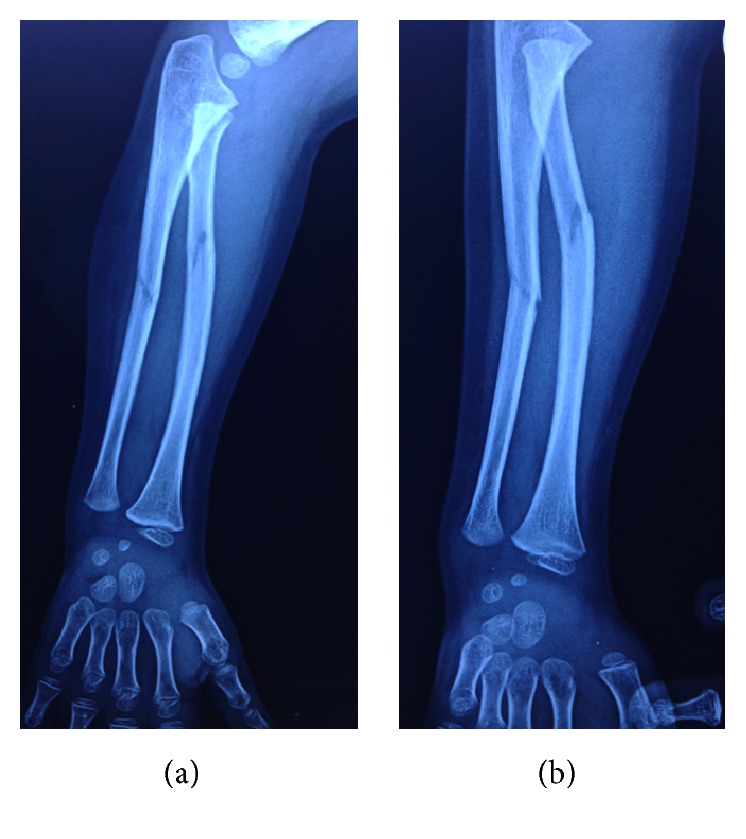
Oblique forearm radiographs taken in outside clinic revealed both bone forearm fractures and no anterior displacement of radial head.

A 5-year-old patient was sent to our orthopaedic outpatient clinic from another emergency clinic with a diagnosis of a both-bone forearm fracture. It was reported that he fell on his hand. His arm was stabilized with a long arm air-cast when he came, but no intervention of any kind was performed. Existing forearm biplanar radiographs obtained in the outside clinic confirmed the diagnosis (Figure 1).

There were no true elbow radiographs. Closed reduction and cast application were performed. Control radiographs revealed a lateral radial head dislocation. Operative intervention was planned and performed.

### 2.1. Investigations

**Figure 2:**
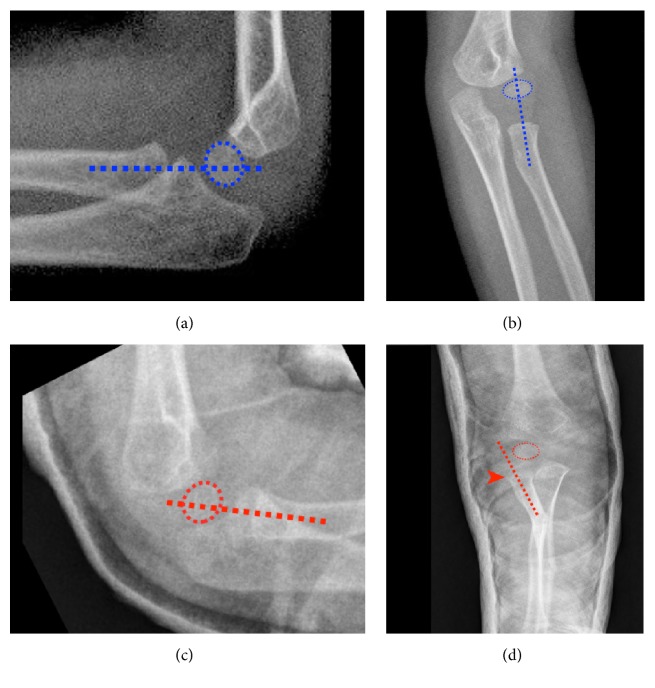
AP (a) and lateral (b) views of the healthy side with radiocapitellar lines. AP (c) and lateral (d) views of the injured side with radiocapitellar lines after closed reduction. Arrow head shows laterally dislocated radial head.

Existing radiographs showed no true elbow views and forearm radiographs were taken under suboptimal conditions (Figure 1). After our closed reduction, biplanar forearm and elbow radiographies were taken of both arms (Figure 2).

### 2.2. Differential Diagnosis

The patient was diagnosed first with a both-bone forearm fracture. Existing radiographs (Figure 1), although taken under suboptimal conditions, revealed no malalignment of the radiocapitellar line, a line drawn along the long axis of the radial neck, which should bisect the capitellum on all projections [1]. Therefore an anterior radial head dislocation was excluded.

**Figure 3:**
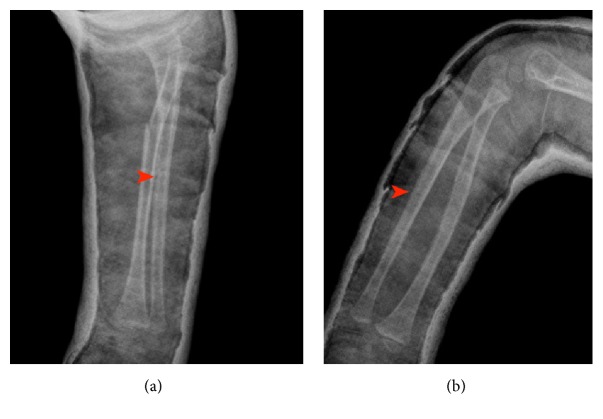
Anatomic reduction of the ulnar fracture in AP (a) and lateral (b) views. Arrow heads show hardly recognizable ulnar fracture lines.

In case of a Monteggia fracture-dislocation, both bone forearm fractures exist with anterior displacement of radial head according to the Bado classification [2]. Radiographs taken after closed reduction and cast application, however, revealed a lateral dislocation of the radial head (Figure 2). The ulna was reduced (Figure 3). Operative intervention was planned.

## 3. Treatment 

The ulna was anatomically reduced after closed reduction (Figure 3). An intramedullary Kirschner wire was implanted into the radius. Intraoperative fluoroscopy images revealed the successful reduction of the radial head and proved the already reduced ulna to be stable. No surgical intervention for stabilization of ulna was planned and a long arm cast was applied.

**Figure 4:**
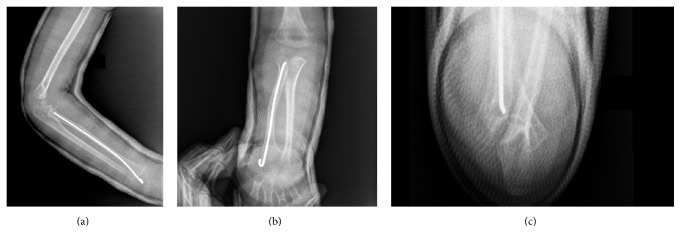
Postoperative radiographs show the radial head and anatomic ulnar reductions in lat (a), AP (b), and axial (c) views.

Postoperative radiographs proved the success of reduction (Figure 4).

The long arm cast was removed after 3 weeks and active motion was allowed thereafter. At the 6th postoperative week, the K-wire was removed operatively.

**Figure 5:**
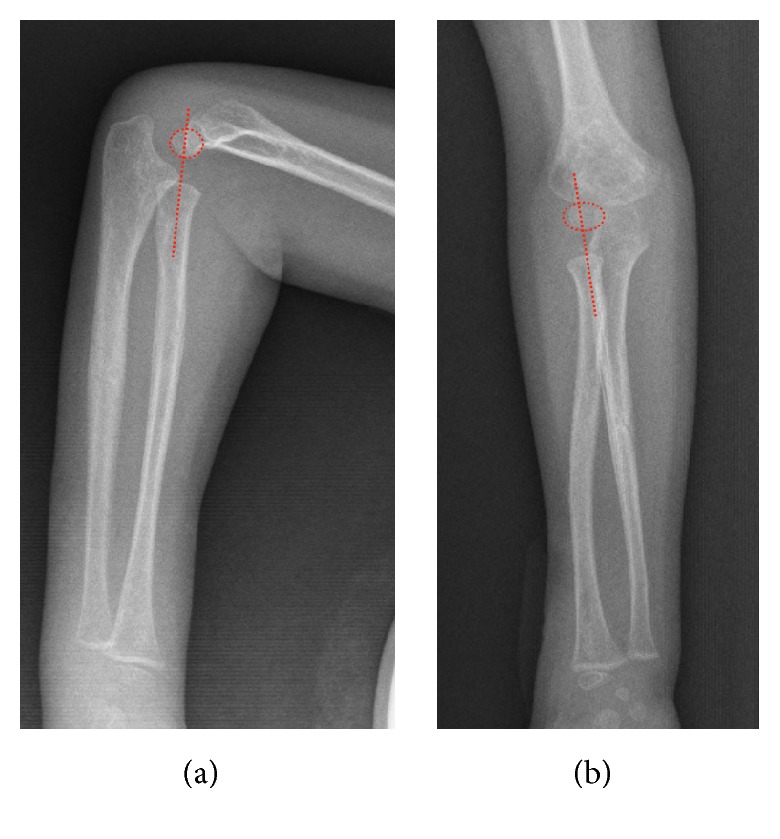
Radiographs after K-wire removal show successful union and reduction at the 6th week.

**Figure 6:**
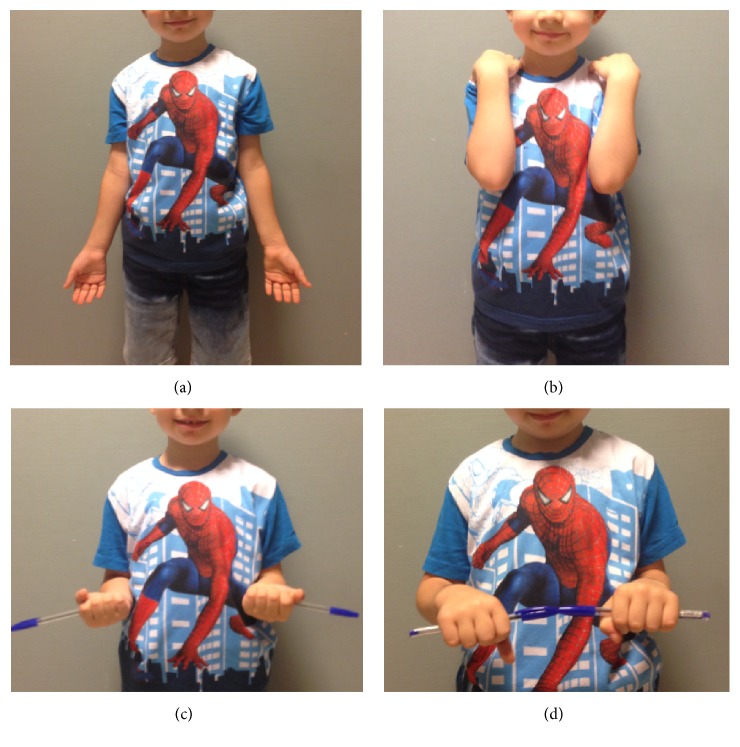
Clinical photos show full ROM of elbow and forearm rotation at the 8th week.

Two weeks after removal of the K-wire, radiographs revealed successful union of both bones (Figure 5). His elbow flexion and extension and forearm pronation and supination were unrestricted (Figure 6).

## 4. Outcome and Follow-Up 

The patient was discharged on postoperative day 1. He was followed for 8 weeks at 2 weeks' intervals.

At the 6th week K-wire was removed.

He was examined on the 8th week, revealing unrestricted ROM of elbow and forearm (Figure 6).

## 5. Discussion 

This case highlights the importance of thorough clinical and radiological evaluation of pediatric patients with both bone forearm fractures. Failure to obtain and interpret good-quality radiographs properly is the most common problem of Monteggia fractures described in the literature [3]. Our case is another proof for this description. Adequate AP and lateral elbow radiographs must be taken in all pediatric forearm fractures of both bones.

This case reports a fracture-dislocation pattern that was not previously described in the literature. According to the Bado classification, pediatric Monteggia fractures are classified by direction of the radial head dislocation and type of forearm fracture [2] (Table 1).

There are also Monteggia equivalent lesions, which describe fracture-dislocations equivalent to each subtype of the Bado classification. The direction of the radial head dislocation is the primary condition to classify equivalent injuries. Hence a forearm fracture of both bones is not always classified as type IV but rather in accordance to the direction of the radial head dislocation (Table 1). The unusual fracture-dislocation pattern in this case, namely, the lateral dislocation of the radial head and fracture of ulnar shaft, is a candidate to be named as a Monteggia type 3 equivalent lesion.

Treatment is simple when diagnosed, but clinical consequences of delayed Monteggia fractures are dire as reported in the literature. Delayed diagnosis is still reported to constitute an important problem [4]. Undefined Monteggia lesions, like the one described in this case, might have contributed to missed diagnoses in the past. Therefore it is important for clinicians to critically evaluate and interpret radiographs in a patient with a forearm fracture of both bones.

In addition to the diagnostic features, we highlight a unique treatment method. Some authors believe that the purpose of treatment is to obtain an anatomically reduced radial head, which can often be accomplished with less than anatomic reduction of the ulna [3]. Others claim that the success of the treatment depends on an anatomic reduction and stabilization of the ulna [4]. In our case, although the ulna was reduced anatomically, the radial head was still dislocated laterally. We decided to insert an intramedullary nail in the radius and check the stability intraoperatively. This method provided an acceptable stabilization without the need for fixation of the ulna fracture.

## Figures and Tables

**Table 1:** Lateral radial head dislocation with concomitant both-bone forearm fracture fits in the Bado classification as a type 3 equivalent lesion, which has never been described [2].

Bado classification			
	Radial head dislocation	Forearm fracture	Equivalents
Type I	Anterior	Ulnar with anterior angulation	Isolated radial head dislocation Isolated radial neck fracture Diaphyseal ulnar fracture with radial neck fracture Diaphyseal ulnar fracture with fracture of the proximal third of radius Diaphyseal ulnar and olecranon fracture with anterior dislocation of the radial head Diaphyseal ulnar, posterior dislocation of the elbow ± fracture of the proximal third of radius

Type II	Posterior	Ulnar with posterior angulation	Epiphyseal fracture of dislocated radial head

Type III	Lateral	Proximal ulna	—

Type IV	Anterior	Both bones	—

## References

[1] Kunkel S., Cornwall R., Little K., Jain V., Mehlman C., Tamai J. (2011). Limitations of the radiocapitellar line for assessment of pediatric elbow radiographs. *Journal of Pediatric Orthopaedics*.

[2] Rehim S. A., Maynard M. A., Sebastin S. J., Chung K. C. (2014). Monteggia fracture-dislocations: a historical review. *The Journal of Hand Surgery*.

[3] Herring J. A., Ho C., Herring J. A. (2014). Upper extremity injuries. *Tachdjian's Pediatric Orthopaedics*.

[4] Dolan M., Waters P., Green N. E., Swiontkowski M. F. (2009). Fractures and dislocations of the forearm, wrist, and hand. *Skeletal Trauma in Children*.

